# Self-interpreted narrative capture: A research project to examine life courses of Amerasians in Vietnam and the United States

**DOI:** 10.1177/2059799119863280

**Published:** 2019-07-12

**Authors:** Sabine Lee, Susan Andrea Bartels

**Affiliations:** 1 Department of History, University of Birmingham, Birmingham, UK; 2 Department of Emergency Medicine, Queen’s University, Kingston, ON, Canada

**Keywords:** Children born of war, Vietnam War, mixed methods narrative capture, Amerasian, SenseMaker

## Abstract

When American troops withdrew from Vietnam in April 1975, they left behind a large number of children fathered by American GIs and born to local Vietnamese women. Although there is some documentation of experiences of GI children who immigrated to the United States, little is known about the life courses of Amerasian children who remained in Vietnam, and no comparative data has been collected. To address this knowledge gap, we used an innovative mixed qualitative – quantitative data collection tool, Cognitive Edge’s SenseMaker^®^, to investigate the life experiences of three specific cohorts of GI-fathered children from the Vietnam War: (1) those who remained in Vietnam, (2) those who immigrated to the United States as babies or very young children and (3) those who immigrated to the United States as adolescents or adults. The current analysis reflects on the implementation of this mixed-methods narrative data collection and self-interpretation tool as a research methodology in Vietnam and the United States and outlines some of the challenges and lessons learned including recruitment of a hard to reach population, low response rates in the United States and feasibility of using such narrative capture to conduct such research in the United States and in Vietnam.

## Introduction

When American troops, after years of engagement and conflict in Vietnam, withdrew in April 1975, they not only left behind a war-torn country but also personal and sometimes intimate relationships, a large number of which had resulted in children being born (McKelvey,1999). These American GI-children, born to Vietnamese women, are one of many groups of children born of war (CBOW), defined as children fathered by foreign soldiers and born to local mothers (Lee and Mochmann, 2015: 18–19). Recent research (Carpenter, 2007; Ericsson and Simonsen, 2005; Lee, 2017; Seto, 2013) suggests that CBOW were exposed to significant childhood adversities and often suffered from stigmatisation and discrimination, (Glaesmer et al., 2012), but the evidence base beyond specific CBOW groups during and after the Second World War (Ericsson and Simonsen, 2005; Lee, 2011; Mochmann and Larsen, 2005; Muth, 2008; Stelzl-Marx and Satjukow, 2015; Virgili, 2009; Westerlund, 2011a, 2011b) remains limited.

Unlike many other groups of CBOW, the Vietnamese GI-children, born in the late 1960s and early 1970s at the height of the conflict, did receive some political attention. As the war had been an ideological as much as a military or political conflict, ideological divisions were expected to continue long after the defeat of the South Vietnamese Army by the VietKong and withdrawal of the American forces. The US government anticipated a demonisation of all things American and significant hardship for those in Vietnam with clear links to the American enemy, above all children visibly identifiable as offspring of American GIs. Thus, the United States was cognisant of the existence of American GI-children in Vietnam, who came to be derogatorily referred to as *Bui Doi* (Dust of Life; Taylor, 1988; U.S. General Accounting Office, 1994: 1–3), and some Vietnamese Amerasians were evacuated at the end of the war in April 1975, as part of the so-called Operation Babylift, a US-government-backed initiative that saw the transport of several thousand young children to America, Canada and Europe (U.S. Agency for International Development, 1975).

Subsequently, as part of the Orderly Departure Programme of 1979 (Kumin, 2008) and the Amerasian Immigration Act (1982), a further 6000 Amerasians and 11,000 of their relatives immigrated to the United States. In the most recent attempt to ‘bring home’ the children of American GIs born in Vietnam, the so-called American (or Amerasian) Homecoming Act of 22 December 1987 allowed Amerasians (defined as children of American citizens born between 1 January 1962 and 1 January 1976) and their relatives to apply for immigration to the United States. By 2009, approximately 25,000 Amerasians and between 60,000 to 70,000 of their relatives had immigrated to the United States under the American Homecoming Act (Lee, 2015: ii; U.S. General Accounting Office, 1990). Following these various waves of evacuation and emigration, an estimated 400–500 Vietnamese Amerasians are thought to have remained in Vietnam (Lind, 2016).

Understanding of the life courses and experiences of Amerasians has been patchy, with clusters of research around psychosocial outcomes and mental health pathologies on the one hand (Bernak and Chung 1997; Felsman et al., 1989; McKelvey et al., 1992, 1993; McKelvey and Webb, 1993, 1995, 1996) and explanatory work of their living conditions, often based on anecdotal evidence collected in oral history projects and ego-documents, on the other (Bass, 1997; De Bonis, 1994; Hayslip, 2003; Sachs, 2010; Yarborough, 2006). Even less is understood about Amerasians in Vietnam with no published research about their experiences nor any data collected about their mental and physical health outcomes or socio-economic circumstances. In contrast, some experiences of Amerasians who later immigrated to the United States have been recorded, including early childhood experiences in Vietnam and life courses in America (Lamb, 2009; Long, 1997; Valverde, 1992; Yarborough, 2006). In a 1994 US-based survey, 71% of Amerasians interviewed reported experiences of discrimination in Vietnam, including difficulty in accessing schooling, negative attitudes by teachers, grade discrimination and persistent offensive teasing by peers (U.S. General Accounting Office, 1994: 71).

Post-migration reporting, especially in the media and often around the anniversaries of the Babylift and the Homecoming Act, has tended to emphasise the greater opportunities for Amerasians in the United States (Gaines, 1995a, 1995b; Sachs, 2010; Taylor, 1988; Valverde, 1992). However, ego-documents (De Bonis, 1994; Bass, 1997; Yarborough, 2006, Chapters 7–9) also reveal that integration into the father’s home country was challenging. Significantly, most Amerasians had to abandon their dreams of a family life that included both parents, as only a fraction of Amerasian immigrants managed to contact their American fathers after arriving in the United States (Lamb, 2009) In particular, Amerasian migrants with little schooling, limited English and few transferable skills, as well as those with Afro-American fathers, reportedly found adaptation to American life challenging (Ranard and Gilzow, 1989: 1–3).

The aim of this project is to provide a more nuanced understanding of the challenges faced by Vietnamese Amerasians in both the United States and Vietnam to inform policy recommendations aimed at addressing and mitigating the difficulties they and similar groups of CBOW experience in their mothers’ and fathers’ home countries. To achieve this, we used a research tool called SenseMaker^®^ developed by Cognitive Edge. Its fundamental principle, the collection of self-interpreted narratives, is based on the recognition that storytelling is an important form of human communication used by individuals to make sense of their own and their community’s experiences (Brown, 2006; Koenig Kellas and Trees, 2006) as well as being a useful method for creating individual and collective memories. (Thomson, 2011) Through the narratives recounted in storytelling, people make sense of their personal experiences (Fivush et al., 2011). Using SenseMaker, participants share a story in response to their choice of open-ended prompting questions and this story generates qualitative data in the form of brief narratives collected as audio or text files. After recording a micro-narrative, participants then self-interpret the described experiences by answering a series of pre-defined questions relating to the events in the story and these responses generate the accompanying quantitative data. Based on complexity theory (Burnes, 2005), SenseMaker helps to understand people’s experiences in complex, ambiguous and dynamic situations by using pattern detection software to identify common themes. Using a mixed methods approach, it leverages the ‘wisdom of the crowds’ by collecting a large number of stories to give statistical power while still providing qualitative depth through the accompanying linked narratives. Because there are no responses that can be perceived as obviously better than others, SenseMaker reduces social desirability bias and because participants interpret their own narratives using a series of pre-defined questions, the researchers’ interpretation bias is also reduced. This kind of self-interpreted narrative capture, thus, can offer a more nuanced understanding of complex issues by using indirect prompting questions that tend to elicit more honest and more revealing responses. The authors have no relationship with Cognitive Edge and no conflict of interest around use of SenseMaker.

While SenseMaker has been investigated as a tool for dealing with inherently complex management and evaluation problems (e.g. Gorzeń-Mitka and Okręglicka, 2014; Guijt, 2016; Milne, 2015), less has been written about its application to research involving complex human scenarios. Therefore, we aim to contribute to the fledgling literature that assesses both the opportunities of self-interpreted narrative capture as well as challenges and limitations of the methodology for such research in a policy-relevant setting. In particular, this article explores how narrative capture allows the collection of nuanced self-interpreted stories from Amerasians to investigate the social outcomes for three specific cohorts of GI-fathered children from the Vietnam War: (1) those who remained in Vietnam, (2) those who immigrated to the United States as babies or very young children and (3) those who immigrated to the United States as adolescents or adults. We describe both the browser-based and tablet-based collection of micro-narratives and related quantitative data, while assessing the usefulness of each data collection method among various participant subgroups. Implementation challenges in each of the US and Vietnamese contexts are also presented along with reflections on lessons learned for future research involving CBOW.

## Methods

This cross-sectional, mixed qualitative–quantitative study was conducted in Vietnam and the United States in 2017. Data collection in Vietnam occurred in April and May in collaboration with the Department of Anthropology at the University of Social Sciences & Humanities at the Vietnam National University in Ho Chi Minh City and the Vietnam chapter of *Amerasians Without Borders*, a US-based non-profit organisation of Vietnamese Amerasians who support Amerasians, among others through facilitation of DNA tests in order to support immigration into the United States. Data collection in the United States occurred from February to July in collaboration with the US chapter of *Amerasians Without Borders.*

### Participant recruitment

Individuals from the age of 11 years were eligible to participate. A variety of participant subgroups were targeted for recruitment to capture a wide range of perspectives about the life experiences of Amerasians. These subgroups included Amerasians themselves, mothers of Amerasians, spouses of Amerasians, biological fathers and stepfathers of Amerasians, adoptive parents of Amerasians, children of Amerasians, other relatives of Amerasians and community members where Amerasians live.

Interview sites were chosen purposively based on existing data about where Amerasians were thought to be living. In Vietnam, the chosen interview sites were Ho Chi Minh City, Dak Lak, Quy Nhon and Da Nang. In each of these four study locations, *Amerasians Without Borders* organised group meetings in which members and their relatives were invited to a designated location to meet with the interview team. After the study was introduced to potential participants, consenting Amerasians and their families were asked to privately share a story about the experiences of Amerasians in Vietnam (either a personal story or a story about an Amerasian family member) and to then interpret the story by completing the SenseMaker survey.

In the United States, chosen sites for face-to-face interviews included San Jose California; Portland Oregon; Santa Ana, California and Chicago, Illinois. The interviewers travelled to each of these four study locations to meet participants with whom interviews had been pre-arranged through contacts within the *Amerasians Without Borders* social network. Interviews in Chicago were conducted at the *Amerasians Without Borders* annual meeting in July 2017. A link to the browser-survey offered in the United States was posted on Facebook and Twitter by *Amerasians Without Borders* in addition to being emailed to their members. In the United States, *Operation Reunite* (http://www.adoptedvietnamese.org/avi-community/other-vn-adoptee-orphan-groups/operation-reunite/. Accessed 9 August 2017), an organisation which aims to raise awareness of the Vietnam War and to provide support to Vietnamese war babies brought to the United States and other countries like the United Kingdom, France and Australia at the end of the war, also provided support for data collection in the United States. Its social media platforms were leveraged to share information about the study and to distribute the browser link to Amerasian children who had immigrated to the United States through Operation Babylift.

### Survey instrument

The SenseMaker survey was drafted iteratively in collaboration with an experienced narrative capture consultant and was reviewed by Vietnamese and Amerasian partners. Choosing one of two open-ended prompting questions, participants were asked to share an anonymous story about the life experiences of an Amerasian in Vietnam or in the United States. After sharing the story, participants were asked to interpret the Amerasian’s experience by plotting their perspectives between three variables (triads), using sliders (dyads) or on a graph (stones). Multiple-choice questions followed to collect demographic data and to contextualise the shared story (e.g. emotional tone of the story, how often do the events in story happen, who was the story about, etc.). The survey was drafted in English, translated to Vietnamese and then back translated by an independent translator to resolve any discrepancies. The Vietnamese and English versions of the survey were uploaded to the Cognitive Edge secure server for use in Vietnam and the United States, respectively. Both surveys were reviewed for errors, and corrections were made prior to initiation of data collection.

In the United States, data were similarly collected using the SenseMaker app on iPad Mini 4, but a browser version of the survey was also made available. The browser survey, which was identical to that on the SenseMaker app, was circulated through various social networking platforms of *Amerasians Without Borders* and *Operation Reunite.* The browser survey was introduced in the United States where widespread availability of the Internet allowed the link to be shared with a large number of potential participants, many of whom were thought to be able to access the Internet independently to complete the survey at their convenience.

### Procedure

In Vietnam, the data collection team consisted of eight interviewers from the Department of Anthropology at the University of Social Sciences & Humanities at the Vietnam National University in Ho Chi Minh City and included two faculty members as well as six graduate students. Immediately prior to data collection, all interviewers participated in a two-day training on narrative capture research ethics, use of an iPad, how to approach participants and obtain informed consent, specific survey questions with multiple role-playing sessions, data management, adverse events and programme referrals. In Vietnam, all data were collected on the SenseMaker app using iPad Mini 4. Collected data were stored on the iPad until it was possible to connect to the Internet, at which time it was uploaded to Cognitive Edge’s secure server. During the upload process, data were automatically deleted from the tablet.

In the United States, two interviewers identified through *Amerasians Without Borders* collected data. Both self-identified as Amerasian and received individual training on the above topics immediately prior to data collection. During data collection at the *Amerasians Without Borders* annual meeting in Chicago, they were supported by three fully trained interviewers, including a faculty member, a student and a volunteer. The browser survey used in the United States was posted on Facebook and Twitter by *Amerasians Without Borders* with individuals completing the survey independently and uploading the data directly to the Cognitive Edge secure server.

At each of the pre-selected interview locations, potential participants in each of the targeted subgroups were identified through the social networks of *Amerasians Without Borders.* Interviewers introduced the study using a pre-defined script, and if the individual expressed interest in participating, the interviewer and participant chose a private location that was out of earshot of others. Participants were then asked to tell a story about the experiences of an Amerasian based on their choice of two story prompts. Shared stories were audio-recorded on tablets and participants then responded to a series of pre-defined questions. If the participant was uncomfortable having his/her voice recorded, the interviewer first listened to the participant’s story and then recorded the story in his/her own voice on behalf of and in front of the participant. All participants were asked if they would like to share a second story and therefore the number of shared stories exceeds the number of unique participants. A graduate student oversaw data collection in Vietnam by reviewing uploaded data on a weekly basis and performing quality assurance checks.

### Ethical considerations

All interviews were conducted confidentially and no identifying information was recorded, thus the data were anonymous from the start. Participants were asked not to use actual names or other identifying information in their shared stories, and in the event they did, the name or other identifying information was not transcribed. In the facilitated interviews, informed consent was explained to the participant prior to the interview in either Vietnamese (in Vietnam) or English (in the United States) and was indicated by tapping a consent box on the handheld tablet. In the browser version, participants read the explanations of informed consent in English and clicked the consent box to indicate their willingness to participate. No monetary or other compensation was offered but expenses incurred to travel to the interview were reimbursed and refreshments or a light meal were provided. The University of Birmingham’s Ethical Review Board approved this study protocol (Ethical Approval ERN_15-1430).

### Definitions

For the purposes of this article, ‘Amerasian’ refers specifically to Vietnamese Amerasians born to Vietnamese mothers and GI-fathers during the Vietnam War.

### Analysis

Participants’ responses on the story interpretation (i.e. triads, dyads and stones) generate quantitative data in the form of plots, where clusters reveal widely held perspectives on particular issues. If a large volume of self-interpreted stories is captured, SenseMaker facilitates harvesting the ‘wisdom of the crowds’ and helps to ascertain patterns across various subgroups offering insights into mainstream, alternative and diverse perspectives on a topic of interest. This quantitative data are contextualised and interpreted in conjunction with the accompanying narratives, thus offering a rich mixed methods analysis.

The results presented here are focused exclusively on the implementation of the research in both Vietnam and the United States among three different cohorts of Amerasians. Quantitative and qualitative data will be presented separately.

## Results

In total, 319 self-interpreted stories were collected from 231 unique participants in Vietnam, and 58 stories were collected from 55 unique participants in the United States. A variety of subgroups were included as outlined in Table 1 to provide different perspectives.

**Table 1.** Number of stories and number of unique participants in each subgroup by country.

	Vietnam		The United States	
	Stories	Unique participants	Stories	Unique participants
Amerasian	203	138	41	38
Mother of Amerasian	12	8	0	0
Stepfather of Amerasian	0	0	1	1
Biological father of Amerasian	1	1	2	2
Spouse of Amerasian	52	45	2	2
Child of Amerasian	31	27	1	1
Other family member of Amerasian	17	11	5	5
Community members	3	2	1	1
Community leaders	0	0	1	1
Missing	0	0	3	3
Other	0	0	1	1
Total	319	231	58	55

### Recruitment of a hard to reach population

Earlier documentation suggested that the Amerasians in Vietnam had faced considerable stigmatisation and discrimination as a result of being visibly connected with the American enemy (McKelvey, 1999: 21; Yarborough, 2006: 41). Consequently, we anticipated that it would be challenging to reach the Amerasians in Vietnam for the purposes of this research, and it was unknown if the Amerasians would be willing to talk with the research team about their life experiences. By recruiting through the Vietnam chapter of *Amerasians Without Borders*, however, we were able to interview 231 unique participants, 138 of them Amerasian themselves. Not only were we able to connect with a surprising number of Amerasians and their family members over a 3-week period, but some of the research participants travelled considerable distances to be able to take part in the study. Furthermore, once at the interview site, many of the participants in Vietnam particularly the Amerasians themselves, were eager to tell multiple stories about their experiences as American GI-children, and the stories were quite lengthy sometimes up to 60 minutes in duration. This is significantly longer than the average length of SenseMaker micro-narratives which is approximately 5 minutes. The Vietnam data collection was successful because of *Amerasians Without Borders’s* social networking and because of the trust that many of the Amerasians had in the organisation. However, the fact that the participants travelled for such distances and shared so many details about their personal lives also indicates a strong desire to have their voices and their stories heard.

While recruitment through *Amerasians Without Borders* was critical in allowing us to reach Amerasians in Vietnam, it is also important to note this as a limitation of the study. The Amerasians who were interviewed in Vietnam were mostly members of *Amerasians Without Borders* and therefore presumably receiving support, at least peer support, if not assistance with tracing their biological fathers, filing documentation to immigrate to the United States and so on. The study was unfortunately not able to reach many Amerasians who were not members of *Amerasians Without Borders*, and it remains unknown if their stories about life experiences would have been different.

By not interviewing Amerasian participants in their own communities, we under-recruited community members and relatives of Amerasians (Table 1). However, by having Amerasian participants travel to designated locations to meet the study team and participate in the research, we were able to maximise the number of first-person stories about the experiences of Amerasians in Vietnam.

Self-interpreted narrative capture was well-suited for the collection of rich and nuanced first-person stories of this particular cohort. Due to the participants being ‘in control’ of the story, thus determining which details and the degree of sensitivity to be shared, it was possible for vulnerable participants, including children and older participants to contribute to the study in an ethically acceptable way. This allowed us to gain a transgenerational perspective over three generations of a large number of potentially vulnerable participants. Since no personal or identifying data was recorded, participation among a population that had experienced severe hardship and extreme discrimination was facilitated; it also protected participants when the Vietnamese authorities attempted to obtain identifying information of the study’s participants.

**Figure 1.**
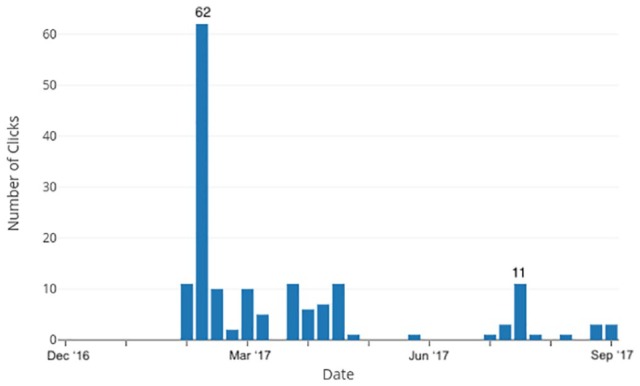
Number of site visits by survey weeks.

US-based Amerasians were originally assumed to be more easily reachable than Amerasians residing in Vietnam. The reason for this was not only their significantly larger numbers, but also their greater visibility, not least due to considerable media attention at anniversary milestones of the Babylift and Homecoming Act. Moreover this group was reportedly well networked through support organisations and social media. While it was possible to make initial contact with some Amerasians in the United States, especially via social networks and on the occasion of the *Amerasians Without Borders* annual meeting in Chicago, the overall response rate was low in the United States, both with the facilitated tablet-based survey and the browser survey. The weeks with the highest number of people visiting the survey site were the week following the initial posting on social media (62 site visits), and after a period of almost complete disengagement in May and June, another spike followed in the week of the *Amerasians Without Borders* annual meeting (11 site visits; Figure 1).

However, survey completion rates remained low throughout. Of the 100 people who visited the online site in the first 6 weeks after it was posted, only six completed the survey.

By the end of the 6-month data collection period, 162 people had visited the site, but only 10 had completed the survey as shown in Table 2.

**Table 2.** Number of completed surveys at certain dates.

Date	18 February	25 February	13 March	20 March	27 March	3 April	17 April	23 April	31 July
Number of surveys (total)	4	4	6	7	7	9	10	10	10

Overall, the narratives captured from participants both in the United States and in Vietnam were relatively long in comparison with most SenseMaker projects. Respondents were inclined to deviate from the story prompt, which asked them to share one specific experience in the life of an Amerasian, and instead they tended to narrate a range of stories and experiences. This impacted on the ability of participants to self-interpret the narrative via the questionnaire, as the answers on occasion related to different aspects of the multi-facetted narratives, which led to some ambiguities. This problem was more pronounced in the case of Amerasians in the United States because they frequently combined narratives of their experiences in Vietnam with those in the United States after immigration.

### Low response rate in the United States

Despite endorsement of the research by leadership of *Amerasians Without Borders*, and enlistment of well-networked Amerasian interviewers, the initial enthusiasm in the United States to participate in the survey was followed by reluctance to engage. In discussion with the research assistants, the following reasons for the low take-up were identified:

1. Scepticism about data use: When Amerasians in the United States became aware that a Vietnamese university was partnering on the project, they expressed doubt about the anonymity and confidentiality of the data. A deep-seated mistrust in any Vietnamese institutional involvement led to reservations vis-à-vis participation because of fear that data would be misused for political purposes.
2. Fragmentation of Amerasian community in the United States: While many Amerasians in the United States are organised in support groups, these are fragmented and differ in focus and support orientation. The different organisations do not always co-operate and sometimes compete with each other, which may have resulted in Amerasians being discouraged from participation in a project that was perceived as being endorsed explicitly by one specific group.
3. Re-traumatisation: A significant number of respondents who initially agreed to be interviewed changed their minds later. In the majority of cases, the reasons appeared to be psychosocial stress linked to recalling their own experiences.
4. Linguistic obstacles: Although many Amerasians in the United States are competent English speakers, some appeared less comfortable with written English, which will likely have impacted the uptake of the online survey.

## Discussion

### Feasibility and utility of self-interpreted narrative capture as a data collection method among Amerasians

#### Vietnam

Self-interpreted story capture proved an efficient data collection method for Amerasians in Vietnam. The story-telling nature of the survey allowed participants to have their voices heard and telling stories about their experiences appealed to the participants, which is evident both in the relatively large number of second and third stories shared and the length of the stories. The immediacy of the quantitative data collection which is available immediately upon uploading the survey to the server is time-effective and by implication cost-effective, as it eliminates lengthy coding processes that are required in more traditional qualitative research.

Debriefing with the Vietnam interview team revealed that a majority of the Amerasian participants in Vietnam were either uneducated or undereducated, and these low literacy and numeracy skills added challenges to the data collection. Plotting one’s interpretation of a story on a one-dimensional or two-dimensional graph (dyad or triad, respectively) requires a significant level of abstraction; this proved difficult for many participants, and interviewers had to re-explain and re-confirm comprehension of the instrument with the participants – in some cases repeatedly. SenseMaker works on the principle of minimum input from the interviewers in order to minimise researcher bias in the data collection; however, this could not always be upheld with this particular study cohort because of the need to intervene in order to ensure the accuracy of participants’ data entry.

While this type of narrative capture suited the participants and led to rich qualitative story data, it was difficult to steer participants away from sharing multi-experience life-course accounts. This posed problems when participants answered the interpretation questions, as it was unclear to which element of the narrative they were referring when they choose their response, especially in cases where the narrative contained a multitude of unconnected experiences.

Despite these limitations, this narrative capture proved an effective data collection method. It was cost-effective, especially given the willingness of large numbers of Amerasians in Vietnam to travel to central interview locations. Furthermore, the narrative element in the survey responded to a need of Vietnamese Amerasians ‘to be heard’ and to ‘tell their stories’. As SenseMaker allows the participant to determine the direction of the survey through complete control over the narrated story, it was possible to collect data about sensitive aspects of individuals’ experience in an ethical way.

While interviewing at central locations facilitated the data collection and allowed for an efficient and cost-effective process, it added a further limitation. By not meeting Amerasians and their families in their local context, the number of family members and community members was limited, and while the target of stories collected from Amerasians themselves was exceeded, the targets for most other groups (spouses, children, mothers and fathers) were missed.

#### The United States

The instrument proved far less effective among the US-based Amerasians. The reasons for this may, however, not be specific to narrative capture but rather to significant differences in the circumstances of the different study cohorts in the United States. In the United States, two distinct groups were identified and targeted: children of the Babylift Operation, and later immigrants who moved to the United States either as part of the Orderly Departure Programme or after the Amerasian Homecoming Act. The first group is known to have integrated well into US society (Lee, 2017: 129–134), and many Amerasians in this cohort do not see themselves as distinct from their ‘American’ peers, however defined. While many among them, having assimilated into American life from an early age, eventually rediscovered their Vietnamese roots (Baden et al. 2012), few saw themselves as part of a distinct and/or disadvantaged group. As such, surveys around specifically ‘Amerasian’ experiences may have held little appeal to them. No Amerasian from the Babylift Operation cohort responded to the different participation requests.

Many individuals belonging to the second group who grew up in Vietnam and emigrated in their late teens initially displayed great interest in study participation but later expressed apprehension about having their information recorded. This apprehension was initially focused on concerns over misuse of data, a fear that was expressed in the context of mistrust of anything connected to the Vietnamese state or government and was potentially heightened by the project’s partnership with an academic institution in Vietnam. Further reasons for non-participation included concerns about confidentiality of the data, unease about inability to express oneself clearly in English and apprehension about navigating the online survey.

While the particular study encountered some of the limitations to the utility of SenseMaker, the use of this innovative methodology was of considerable value in the study of the experiences of Amerasians in the United States and Vietnam. The cost-effective story capture not only allowed the collection of hundreds of stories of unique participants, it also provided the respondents’ own interpretation of their experiences, thus minimising researcher bias, which is an essential advantage especially in politically sensitive study contexts. The rich and nuanced data collected in the project, the only substantial primary source collection capturing the experiences of Amerasians in Vietnam to date, is of immense value for historical and interdisciplinary research into the consequences of the Vietnam War.
